# Faith Cure and Cancer

**Published:** 1891-06

**Authors:** 


### FAITH CURE AND CANCER.

A case of death by cancer under treatment by self-styled Christian Scientists, recently occurred in Jamestown, N. Y. A coroner's jury was impaneled, and gave the following verdict, which should be a warning to "Christian science" doctors :—Mrs. Barrows came to her death from cancer of the breast, on the 8th of May. We believe that contributory to this death was the culpable negligence of Mrs. M. J. Smith and Mrs. E. G. Lovejoy, who were advised of the nature of the fatal malady with which deceased was suffering, and failed to resort to or advise treatment by any of the methods known to medical science. We further believe that W. A. Barrows was also negligent of his duty in not securing medical treatment for his wife when there was reason for believing that she was in need of such treatment.

